# Dynamics of Immune Escape during HIV/SIV Infection

**DOI:** 10.1371/journal.pcbi.1000103

**Published:** 2008-07-18

**Authors:** Christian L. Althaus, Rob J. De Boer

**Affiliations:** Theoretical Biology, Utrecht University, The Netherlands; University of Michigan, United States of America

## Abstract

Several studies have shown that cytotoxic T lymphocytes (CTLs) play an important role in controlling HIV/SIV infection. Notably, the observation of escape mutants suggests a selective pressure induced by the CTL response. However, it remains difficult to assess the definite role of the cellular immune response. We devise a computational model of HIV/SIV infection having a broad cellular immune response targeting different viral epitopes. The CTL clones are stimulated by viral antigen and interact with the virus population through cytotoxic killing of infected cells. Consequently, the virus population reacts through the acquisition of CTL escape mutations. Our model provides realistic virus dynamics and describes several experimental observations. We postulate that inter-clonal competition and immunodominance may be critical factors determining the sequential emergence of escapes. We show that even though the total killing induced by the CTL response can be high, escape rates against a single CTL clone are often slow and difficult to estimate from infrequent sequence measurements. Finally, our simulations show that a higher degree of immunodominance leads to more frequent escape with a reduced control of viral replication but a substantially impaired replicative capacity of the virus. This result suggests two strategies for vaccine design: Vaccines inducing a broad CTL response should decrease the viral load, whereas vaccines stimulating a narrow but dominant CTL response are likely to induce escape but may dramatically reduce the replicative capacity of the virus.

## Author Summary

As a result of their high mutation rate, HIV and its counterpart SIV in non-human primates can evade recognition by the host immune response through the generation of viral variants, the so-called escape mutants. This avoidance of cytotoxic T lymphocyte (CTL) mediated killing seems to be one of the major reasons why virus replication is not controlled effectively. However, it remains difficult to investigate the critical properties of the dynamics of immune escape. To this end, we developed a new computational model of HIV/SIV infection consisting of several CTL clones that can recognize specific parts of viral proteins, i.e., epitopes. The simulations allow us to follow the dynamics of immune escape in detail and help to interpret longitudinal data of HIV/SIV infections. Interestingly, changing the relative sizes of the CTL clones leads to a different evolution of the virus. Instead of reducing the number of infected cells, an alternative strategy of vaccine design could be to reduce the replicative capacity of the virus that might have implications for disease progression.

## Introduction

HIV infection in humans and SIV infection in non-human primates is not cleared by the host's immune system. However, there is partial control of virus replication that is mainly attributed to the cytotoxic T lymphocyte (CTL) response [1],[2]. Particularly the evolution of immune escape mutations posing severe fitness costs [3]–[9], suggests that there is a strong selective pressure induced by the CTL response [10],[11].

It is a challenge to acquire longitudinal data on CTL escape since the virus diversity within a host has to be followed over a long phase of chronic infection. Additionally, little data is available during the early phase of infection, especially for HIV where the infection is difficult to diagnose during the first weeks. Asquith et al. [12] analyzed several data sets of immune escape in HIV, and concluded that killing by the CTL response is inefficient in infected humans. More recent studies described more rapid immune escape of SIV, and estimated more efficient killing in non-human primates [13]–[15]. Quantifying the process of CTL escape and estimating rates remains problematic since the underlying process of viral replication might be taken into account [16]. Furthermore, by analyzing escape at single epitopes it is difficult to assess the strength of the total response of different CTL clones recognizing different epitopes. Due to those difficulties it remains a challenge to devise a realistic model of HIV/SIV dynamics and the subsequent CTL escape.

The interaction of viral replication, mutation and selection by different CTL responses appears to be complex. Studies have observed CTL escape very early but also years after primary infection [17]–[19]. Also, it has been shown that the number of epitope-specific CTL responses varies over time [20]–[22]. Additionally, there is much controversy in the field of HIV evolution as to whether stochastic effects play a role in viral evolution within a host [23]. For example, it has been suggested that the waiting time for compensatory mutations prolongs the emergence of escape variants [24]. To better understand these processes, we present a computational model of HIV/SIV infection taking into account the dynamics of a broad cellular immune response targeting different viral epitopes. The simulations allow us to follow the dynamics in detail, and quantify several critical properties to compare them to data.

### Model

**Figure 1:**
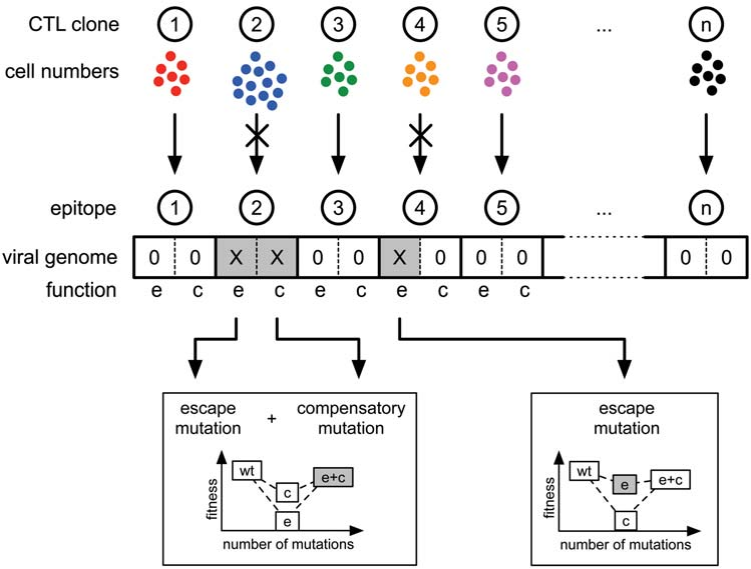
A Scheme of the Computational Model of HIV/SIV Infection. A number of *n* CTL clones can recognize *n* different epitopes and kill the cells infected with virus expressing those epitopes. The virus population can evade recognition from specific CTL clones by acquiring escape mutations (shown as e). Since escape mutations can be associated with a fitness cost in viral replication or infectivity, the virus additionally acquires compensatory mutations (shown as c) that can partially restore the viral fitness.

We develop a computational model of HIV/SIV virus dynamics including a cellular immune response consisting of several CTL clones (Fig. 1). We have *n* different CTL clones recognizing *n* different epitopes derived from viral proteins. On the viral genome we allow two mutations to occur per epitope. One mutation confers escape from recognition by the specific CTL clone. Since escape mutations may be associated with a fitness cost in viral replication or infectivity, a second mutation can at least partially compensate for the fitness loss.

We translate those interactions into a set of ordinary differential equations (ODEs) and add stochastic events for viral mutation. Classically, the processes of infecting target cells or the killing of infected cells by CTLs have been described with simple mass-action terms [25],[26]. For instance, a previous study already described antigenic escape from CTL clones during HIV-1 infection with a simple mathematical model [27]. Recently, we developed terms describing a density-dependent infection that results in a better description of the dynamics of acute infection (i.e. less oscillatory) of the viral load and the immune response [28]. Moreover, the interaction of infected cells with effector CTLs, both for proliferation of effector cells and the killing of infected cells, were assumed to saturate according to Michaelis-Menten kinetics. Here, we integrate those interaction terms to devise a new virus dynamics model consisting of several CTL clones that is described by the following differential equations:
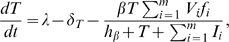
(1)

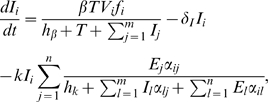
(2)

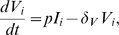
(3)

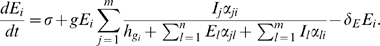
(4)


**Figure 2:**
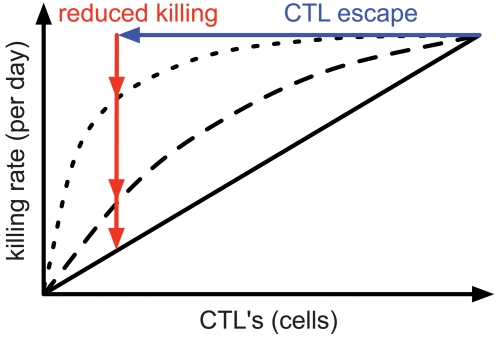
Killing Rate of an Infected Cell as a Function of the Number of CTLs. For killing following mass-action dynamics, the killing rate is linearly increasing with increasing number of CTLs (straight line, *h_k_* = 10^12^). However, if CTL clones compete with each other to kill infected cells, a saturation effect occurs according to Michaelis-Menten kinetics (dashed (*h_k_* = 10^9^) and dotted (*h_k_* = 10^8^) line). After a virus escapes recognition from a single CTL clone (blue arrow), the killing of infected cells is reduced differently depending on these functions (red arrows).

Non-infected CD4^+^ target cells *T* are produced at a rate of *λ* cells per day, die at a rate *δ* *_T_* and can become infected by virus particles of type *V_i_* with fitness *f_i_* at a maximal rate of *β* per day. Target cell availability for virus particles is density dependent, as the infection rate per virus particle is saturating over the total number of CD4^+^ cells (non-infected and infected). After infection of a target cell, reverse transcription occurs during which the virus can mutate with a probability of *μ* per position (for further details see *Methods*). Having two positions to mutate per *n* epitopes, the number of different viral variants, *m*, is maximally *2^2n^*. Infected cells *I_i_* die at a rate of *δ_I_* per day, and are cleared by those CTL clones that can recognize an epitope on its surface. The matrix *α_ij_* defines the topology with which the CTL clone *E_j_* recognizes epitopes presented on the surface of the infected cell of type *i*, and contains either 1 (recognition) or 0 (no recognition). Following Michaelis-Menten kinetics, the CTL clones compete with each other for clearance of infected cells (Fig. 2). When 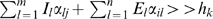, an infected cell is killed at a maximal rate of *k* per day. With increasing *h_k_*, we approach mass-action kinetics for the killing, i.e. when 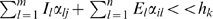 cells are killed at a per capita rate of 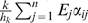 per day. Since the dynamics of virus particles *V_i_* is much faster than that of the cell populations [29], we assume a quasi-steady-state for the virus particles and set *V_i_* = *pI_i_*/*δ* *_V_* [30]. ‘Naive’ CTLs *E_i_* are produced at a rate of σ cells per day. If they recognize antigen produced by *I_j_* (i.e. *α* *_ji_* = 1) they proliferate at a maximal rate *g* per day and die at a rate *δ* *_E_*. Upon infection, virus replicates rapidly and typically 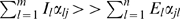. Therefore, CTL effector cells are produced at a half-maximal rate when 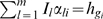. To account for different avidities for the different CTL clones *E_i_* we draw 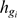 from a uniform distribution. Once the total number of CTL effector cells is high, 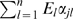 becomes important and we get inter-clonal competition between the CTL effector cells. An overview of the parameters is given in Table 1. For a more detailed description of the model see *Methods*.

**Table 1:** Parameters Used for Simulations of HIV/SIV Infection.

Parameter	Value	Explanation and reference
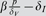	1.5 d^−1^	Initial viral growth rate of 1.5 d^−1^ [36].
*g*	1.0 d^−1^	Maximal CTL proliferation rate [33].
*λ*	2×10^7^ cells d^−1^	Source of CD4^+^ target cells, tuned to obtain an infected cell count between 10_7_ and 10^8^ during the chronic phase [32].
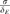	10^3^ cells	Maintains a small number of 10^3^ ‘naive’ cells per CTL clone in absence of infection.
*δ* *_T_*	0.01 d^−1^	Natural death rate of CD4^+^ target cells *T*.
*δ* *_I_*	0.1 d^−1^	Virus-induced death rate of infected cells (includes δ*_T_*).
*δ* *_E_*	0.01 d^−1^	Death rate of cytotoxic T lymphocytes [41],[42].
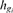	[1.0, 5×10^8^] cells	Uniform distribution of avidities for the CTL clones. Generates a few dominant (low 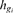 ) and many sub-dominant (high 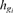 ) CTL clones (see Fig. 6).
*k*	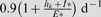	Assumes a maximal death rate of infected cells of 1.0 d^−1^ [37] with CTLs accounting for 90% of infected cell death at the steady-state.
*h_k_*	10^8^–10^12^ cells	Allows killing of infected cells to follow Michaelis-Menten kinetics (small *h_k_*) or to approach mass-action dynamics (large *h_k_*).
*μ*	3×10^−5^	HIV-1 mutation rate per nucleotide [50].
*n*	20	Approximate number of epitopes that are recognized by the CTL responses [21],[40].

## Results

### Sequential Escape from Several CTL Clones

The most surprising phenomenon of CTL escape in HIV/SIV is the time scale at which it occurs. Selection of escape variants has been found to happen very early after acute infection, but also late after years [17]–[19]. Additionally, it has been shown that escape can occur sequentially [31]. Although it has been suggested that compensatory mutations delay the appearance of escape variants, it is still unclear why escape variants would occur so late when the CTL clones recognizing the epitope have been present since acute infection.

**Figure 3:**
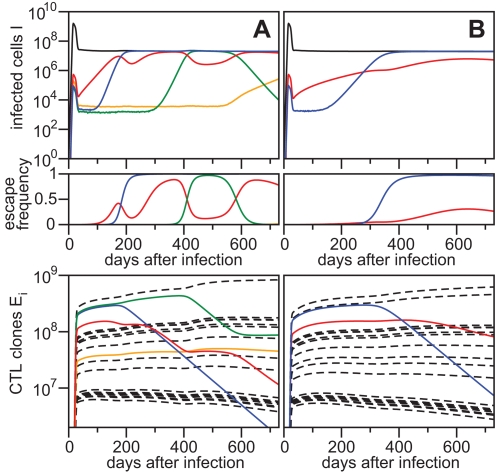
Immune Escape during the First Two Years after Infection. In the top panels, the total number of infected cells (black line) is shown together with the emerging escape mutants (colored lines). Escape variants expressed as a frequency of the total viral population are given in the middle panels. These variants can fluctuate in frequency (e.g. red line) and, after dominating the viral population, revert back to wild-type (e.g. green line). In the bottom panels, a number of CTL clones proliferate upon infection (full and dashed lines) but can slowly disappear after the virus population escapes recognition (full colored lines). Starting with the same CTL repertoire, more escapes occur when killing of infected cells approaches mass-action dynamics (*h_k_* = 10^12^, shown in A) compared to Michaelis-Menten kinetics (*h_k_* = 10^9^, shown in B).

Our model describes the virus dynamics of an HIV/SIV infection and the subsequent immune escape in a very realistic manner (Fig. 3). Many escapes occur widely spaced out in time and we observe that their appearance is determined by the dynamics of the different CTL clones. Starting with the same CTL clone repertoire, Fig. 3A represents a simulation where killing of infected cells approaches mass-action dynamics (*h_k_* = 10^12^). Fig. 3B shows a simulation where killing follows Michaelis-Menten kinetics and CTL clones compete for killing of infected cells (*h_k_* = 10^9^). In both simulations, the total number of infected cells peaks a few weeks after infection and reaches a set-point level of around 10^7^ to 10^8^ cells (black line in top panels) [32]. The colored lines represent the amount of virus-infected cells containing an escape mutation at a specific epitope. Due to the large actual population size of infected cells and the high mutation rate, all single escape and compensatory mutants are produced rapidly during the acute phase of the infection and are maintained in a mutation-selection balance. Some escape variants are being selected early, and replace the wild-type variant at this epitope (e.g., blue line). However, many escapes occur late, despite their early presence in the virus population.

The dynamics of the CTL clones *E_i_* are depicted in the bottom panels. Upon infection, the clones become stimulated depending on the parameter 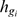 defining their avidity, which generates a single or a few dominant CTL clones and many sub-dominant clones. Escape preferentially occurs from dominant clones that more efficiently kill infected cells. However, due to a severe fitness cost for the escape mutation, there is no escape from the most dominant CTL clone in this particular simulation run. When an escape variant replaces the wild-type variant, the CTL clone looses antigenic stimulation and declines. Because of inter-specific competition between CTL clones, previously sub-dominant clones can increase in size, increasing the selection pressure for the epitopes they recognize.

**Figure 4:**
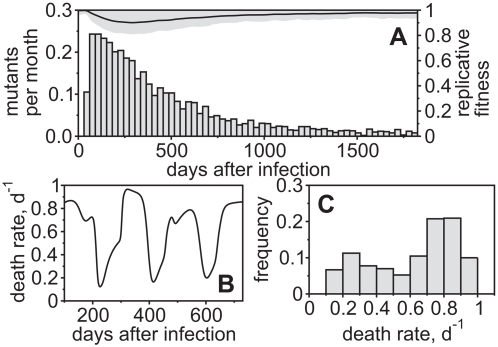
Distribution of Immune Escape over the Course of Infection. (A) Viral escapes over time given as an expected number of escapes per month (average of 1000 simulations). It can be seen that most escapes occur during the first year after infection (acute phase) and fewer afterwords (chronic phase). The straight line shows the replicative fitness of the viral population as an average over all simulation runs (standard deviation is given by the gray area). The time of escape is measured when an escape variant breaches a frequency of 50% of the total viral population for the first time. (B) Time-plot of average infected-cell death rates during the chronic phase of infection. The graph shows one simulation representing a single patient. (C) Histogram of the death rates that are bound between 0.1 d^−1^ and 1.0 d^−1^. For (B) and (C), 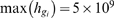. For all figures, *h_k_* = 10^12^, i.e. killing follows mass-action dynamics.

It has been suggested that many escapes occur early, i.e. during the decline phase in viral load after the peak of infection and that they potentially prevent clearance of HIV/SIV. However, in our simulations immune escape does not occur before the set-point level is reached around two to three months after infection (Fig. 4A). This is because the CTL clones only become effective during the decline phase of virus after the peak of infection [28],[33]. Due to a transient CD4^+^ target cell depletion [34]–[36], there is not enough viral replication for escape variants to increase in frequency during this phase. Therefore, escape variants become selected only after the set-point is approached. Nevertheless, many escapes occur during the first months after set-point levels have been attained. After about two years, the virus population stabilizes as the ‘easy’ escapes have been done, the replicative capacity is partially restored and only few escapes are expected to appear later during infection. However, it is important to note that Fig. 4A shows only the first appearance of escape variants. Some of those variants will start to fluctuate in frequency or revert back to wild-type and possibly re-emerge at a later time point. This has implications for the analysis of longitudinal data. If an escape is found to happen late it does not necessarily mean that it had not been selected earlier during infection

Infected-cell death rates from different patients have been found to be close to a normal distribution with a mean of 0.45 d^−1^ [37]. We show that variation of the average infected-cell death rate is also expected to occur within a patient due to the transient decrease of the death rate during an immune escape from CTL mediated killing (Fig. 4B and 4C). However, the death rate does not necessarily decrease over time, i.e. the half-life of infected cells is not expected to increase with disease progression.

### Rates of Escape

Escape variants not only appear at different times during infection but also with different rates. The rate at which an escape variant replaces the wild-type, the so-called escape rate, is determined by the balance between the evaded rate of killing and the fitness cost of the escape mutation [12],[38]. Furthermore, the heterogeneity of the wild-type and the escape variant population at all other epitopes can lead to a different selection induced by other CTL clones. In general, we can define the escape rate as the ‘escape variant growth rate - wild-type growth rate’. A recent study quantified this process and concluded on the basis of slow escape rates and minimal fitness costs that killing of HIV-1-infected cells is inefficient in humans [12]. Unfortunately, the available data sets do not allow us to follow the process of escape in detail. In contrast, our model provides a unique tool to follow the dynamics and analyze rates of escape and link those to rates of killing.

**Figure 5:**
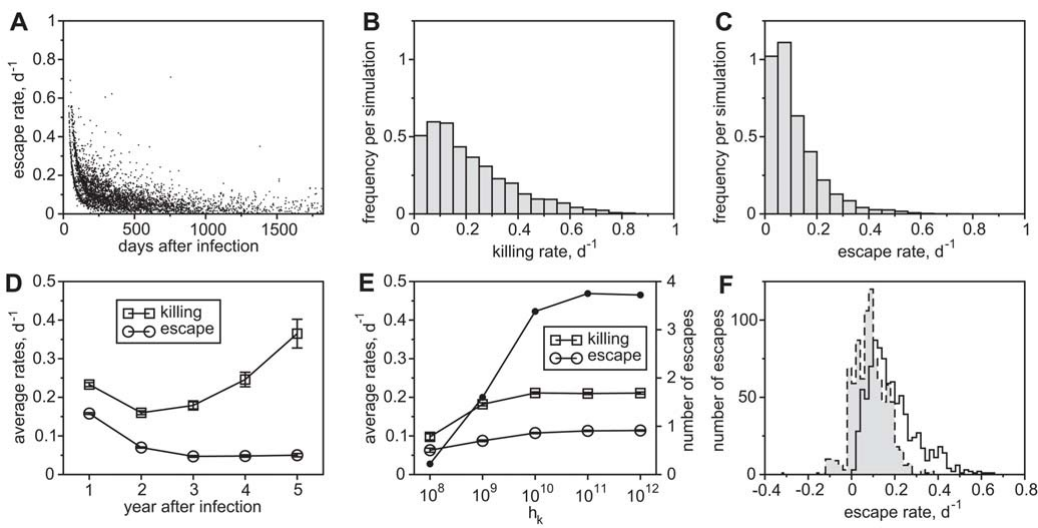
Rates of Killing and Escape. (A) The emergence of escape and the corresponding rates over a time course of 5 years of infection. (B) Given those escapes, the distribution of killing rates given as an average frequency per simulation run. (C) The distribution of escape rates given as an average frequency per simulation run. (D) Average killing and escape rates per year after infection. (E) The number of escapes during 5 years of infection (filled circles) and the average rate of killing and escape as a function of *h_k_* (open symbols). (F) Estimated rates of escape by artificial virus sampling. The two histograms show the distribution of the estimated (gray bars) and the true escape rates (black line). Mean of the estimated rates is 0.09±0.08 d^−1^ and the mean of the true rates is 0.19±0.11 d^−1^. All graphs represent data from 1000 simulation runs. The rates are measured when the frequency of the escape variant is 50%. Means are given with standard errors since standard deviations are usually very large. *h_k_* = 10^12^ if not otherwise indicated.

Fig. 5A shows the distribution of escape and the corresponding rates that occur in 1000 simulation runs over five years of infection. The histograms of killing and escape rates are shown in Fig. 5B and 5C, respectively. Although the total killing by all CTL clones is high (0.9 d^−1^, see Table 1), most individual killing rates are below 0.4 d^−1^, and most escape rates are below 0.2 d^−1^. The lower rates of escape are due to the fitness cost of escape mutations that cannot totally be restored by compensatory mutations. Interestingly, the rates of escape are not distributed equally over the time of infection (Fig. 5A). We therefore calculate the mean of killing and escape rates per year after infection (Fig. 5D). It can be seen that escape rates decrease late during infection and reach relatively low values. The corresponding killing rates decrease after the first year of infection and dramatically increase late during infection. This result shows a) that the selection strength of CTL clones can increase after there has been escape from other clones and b) that escape against efficient CTL clones can be associated with a dramatic cost in viral fitness that slows down the selection of the escape variant.

In Fig. 5A–D we assumed mass-action kinetics for the interaction of CTLs to kill infected cells, i.e., we set *h_k_* = 10^12^. However, when CTL effector cells form a complex with infected cells before delivering their lethal hit, the killing should follow Michaelis-Menten kinetics [28],[39]. We analyzed the influence of a saturating killing term using several lower values of *h_k_* (Fig. 5E). Although the maximal total killing is constant (see Table 1), we can see that killing and escape rates decrease if the model approaches Michaelis-Menten kinetics. Moreover, escape occurs relatively frequent for high values of *h_k_* (mass-action) but rarely for low values of *h_k_* (saturation) (black dots).

To estimate rates of escape from *in vivo* data one has to obtain ratios of escape variants to the wild-type. However, a problem arises when escapes are too rapid to be followed by the relatively long sampling intervals [13]. This shortcoming can lead to an underestimation of the rate of escape. To analyze this effect we measure the frequency of an escape variant at four time points (100, 150, 200 and 250 days after infection) and estimate the escape rate as described in reference [12] (see also *Methods*). Fig. 5F shows the distribution of the estimated escape rates (gray bars) and the true escape rates (black line). Even though the sampling intervals were relatively short (50 days) it can be clearly seen that the estimated rates are generally lower, with a mean of about 50% of the true rates.

Our analysis shows several properties of the process of escape. Even though the total killing induced by all CTL clones is high, escape rates are expected to be slow. First, escape rates slow down due to the acquired fitness cost in viral replication, especially during later phases of infection. Secondly, if killing of infected cells follows Michaelis-Menten kinetics, the rates of escape are decreased. In addition, estimating rates of escape from ratios of escape variants and wild-type virus is likely to lead to an underestimation of the true rate. These findings highlight that slow escape rates do not necessarily infer low killing rates.

### Immunodominance and Viral Evolution

**Figure 6:**
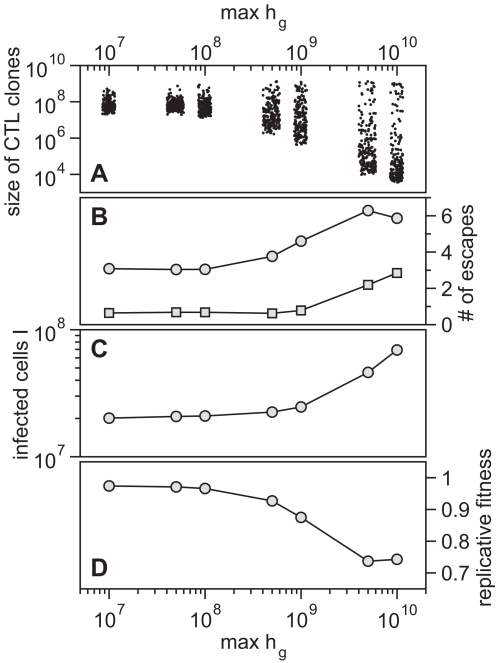
Influence of Immunodominance on Viral Evolution. (A) The distribution of CTL clones plotted as a function of 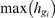. Lower values of 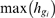 yield a broad repertoire of CTL clones that are similar in size whereas for higher 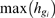 the degree of immunodominance increases. The dots represent the size of CTL clones for 10 simulations at 50 days after infection. Noise is added on the horizontal axis for better visibility. (B) Escape is more frequent for a higher degree of immunodominance. The numbers of escape variants that have occurred within 5 years of infection are shown as circles. As many escapes start to oscillate or revert back to wild-type, the number of escapes that are above 50% in frequency at 5 years after infection is shown as squares. (C) Infected cell numbers increase with increasing immunodominance. (D) The replicative fitness of the virus decreases with increasing immunodominance. Numbers are given after 5 years of infection and represent averages from 1000 simulation runs.

CTL responses during HIV infection generally consist of several CTL clones recognizing many different epitopes. The size of those CTL clones can differ substantially resulting in dominant and sub-dominant responses [21],[40]. In our model, the relative size of a CTL clone depends on how well it can recognize viral antigen, and is defined by 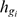. By changing 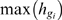 we can change the sizes of the CTL clones relative to each other. For high 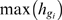 we get strong immunodominance with a single or few dominant clones and many sub-dominant clones, whereas low values of 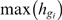 result in CTL clones that are very similar in size (Fig. 6A). Note, that the total killing induced by the sum of all CTL clones remains constant (0.9 d^−1^) and is independent of 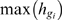.

To investigate the effect of immunodominance we run simulations for different values of 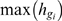. Strong immunodominance, where single CTL clones can induce a strong selection pressure on the viral population, leads to more frequent escape (Fig. 6B). Concurrently, viral replication is controlled less efficiently and the number of infected cells increases with increasing immunodominance (Fig. 6C). However, viral escape can be associated with a fitness cost. Even though the number of infected cells increases, the replicative capacity of the viral population is reduced substantially (Fig. 6D).

## Discussion

The interaction of the HIV quasispecies with the CTL response appears to be complex which makes the analysis of experimental data difficult. Mathematical and computational models have been helpful to investigate how different processes influence each other. For example, a previous model described how shifting immunodominance and antigenic oscillations can occur during HIV infection [27]. Now, with a model incorporating multiple CTL responses together with escape and compensatory mutations, we show that the dynamics of the CTL clones are sufficient to explain the sequential and late occurrence of escape variants. Upon loss of antigenic stimulation, CTL clones disappear slowly [41],[42]. Concomitantly, other CTL clones can increase in size and induce more efficient killing that leads to further escape that is sequentially distributed over many years after infection. Interestingly, the outgrowth of escape variants does not occur earlier even though they are always present in the viral population. It has been suggested that a small effective population size of HIV-infected cells is responsible for the late production and selection of escape variants [43]. However, a recent study argues that stochastic effects play a minor role for the appearance of deleterious and beneficial mutations in HIV [23]. Also, our simulations show that viral evolution is fairly deterministic. The model allows us to simulate two identical ‘patients’ that are infected with the same virus and have the same CTL repertoire. In that case, the stochastic generation of the viral variants results in a slow increase in variation over years (results not shown). For example, the emergence of a certain escape variant is predictable during the first years after infection but becomes more variable later on. This is in line with a recent study where concordant evolution of HIV has been observed in mono-zygotic twins early during infection but more variation has been shown at later stages of the infection [21].

Another explanation for the late appearance of escape variants that has been put forward is the waiting time for compensatory mutations [24]. Our simulations show that single compensatory mutations are expected to be present in the viral quasispecies and therefore should not slow down the emergence of escape. However, if more compensatory mutations are needed to restore the fitness loss of an escape variant they can delay their occurrence (results not shown). Due to epistasis however, the fitness interactions of several mutations are highly complex and therefore simulation results depend mainly on a presumed fitness landscape. Therefore, we propose a way to test the two hypotheses for the late appearance of escape variants. First, if the delay was due to the waiting time for compensatory mutations, late escapes would be associated with more compensatory mutations than early escapes. If on the other hand, the dynamics of CTL clones mainly determines the late appearance, CTL clones where escape has been detected late should have increased in size relative to the other CTL clones in the period before. Both of these hypotheses can be tested by analyzing longitudinal data of HIV/SIV infections.

Our simulations proved to be useful to analyze the process of escape. We conclude that, although killing can be very efficient, escape rates are expected to be low. The association with a fitness cost and the way how CTL clones interact with infected cells critically influence the rates of escape. Hence, it appears to be important to study those interactions to derive realistic killing terms [39]. Furthermore, we have shown that estimating rates of escape is difficult from infrequent sequence measurements. We propose that sequence intervals should be shortened to follow the outgrowth of the variants. Additionally, there might be other reasons that slow down the selection of escape variants. For example, our model does not take into account multiple infected cells. As the fraction of multiple infected cells is large [44], escape variants are likely to appear in cells that are also infected with wild-type virus. As long as wild-type epitopes are presented on the cells surface, the escape variant does not gain any growth advantage compared to the wild-type. As a consequence, the selection of escape variants is expected to slow down.

Immunodominance affects the viral evolution within a host. A higher degree of immunodominance leads to more frequent escape with a reduced control of viral replication but a substantially impaired replicative capacity of the virus. This is interesting as vaccines generally aim to induce a broad CTL response where escape is unlikely to occur. However, a dramatic reduction of the replicative capacity of the virus due to escape could indeed slow down disease progression and/or reduce transmission [45]. Based on these results we identify two strategies for vaccine design: Vaccines inducing a broad CTL response should decrease the viral load whereas vaccines stimulating a narrow but dominant CTL response are likely to induce escape and consequently reduce the replicative capacity of the virus.

The balance between the CTL response and the viral population acquiring escape mutations appears to be a dynamical process over a long period of infection. We have shown that it is important to analyze the kinetics of this process and also the time scale (acute and chronic phase) at which it occurs. More longitudinal data of HIV infections will help to further investigate this process and future research is likely to go into this direction.

## Methods

### Stochastic Events

The set of ordinary differential equations (ODEs) is extended with stochastic events for viral mutation (similar as in [46]). Initially at day 0, infection occurs with the wild-type. Therefore the number of viral variants, *m*(0), is 1. In the following, the ODEs are integrated using the routine odeint from Numerical Recipes [47]. Every time step Δ*t* = 1 day, we approximate the number of cells that have been infected with virus of type *i* during the last time step according to
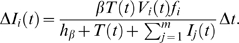
(5)After infection, reverse transcription of the viral RNA occurs during which every position can mutate with the probability *μ*. For the cells being newly infected with virus of type *i*, we calculate the integer number *x_ij_* of cells where the virus mutated into another type *j*. This is done by drawing from the binomial distribution *x_ij_* = *B*(Δ*I_i_*, *μ*) for each of the two positions (escape and compensatory) over a total of *n* epitopes. Then, the infected cell populations are updated accordingly (i.e. *I_i_*  = *I_i_*−*x_ij_* and *I_j_*  = *I_j_*+*x_ij_*). Whenever a previously not existing viral variant *j* is generated, *m*(*t*) = *m*(*t*−Δ*t*)+1, and the set of ODEs is expanded. We also take into account the actual population size of virus infected cells [23]. If the number of infected cells of a certain variant falls below 1, the variant is deleted. Note, that in order for the simulations to be computationally efficient, we only generate the viral variants stochastically but do not include possible fluctuations at small population sizes. However, this hybrid stochastic-deterministic approach is sufficient to keep the variants in a mutation-selection balance. Running the model using the Gillespie algorithm [48], we observed that whenever the different viral variants become selected, their growth approximates the deterministic description from the ODEs. A program of the model was written in C and simulations were run under Linux. The source code can be obtained freely on request from the authors.

### Viral Fitness

Escape mutations in HIV and SIV are likely to confer a fitness cost in viral replication or infectivity [3]–[9]. A recent study by Parera et al. [49] showed that fitness effects caused by random single mutations are approximately uniformly distributed. Therefore, we draw the fitness of a virus containing a single mutation relative to the wild-type virus from a uniform distribution between 0.0 and 1.0. However, the combined effect of an escape mutation together with a compensatory mutation in a single epitope confers a higher fitness that is drawn uniformly between wild-type fitness (*f_wt_* = 1.0) and the higher fitness of the two single variants.

### Estimating Rates of Escape

**Figure 7:**
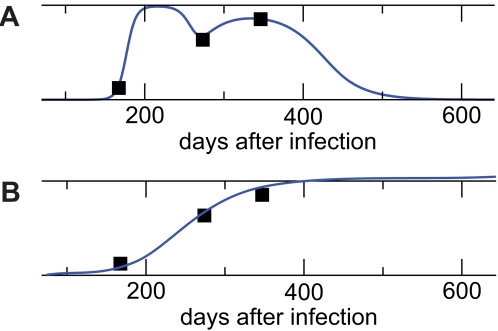
Estimating Rates of Escape. (A) Escape variants often only transiently replace the wild-type variant and oscillate thereafter. Sequence measurements are taken at arbitrary time points (squares). (B) When a model is fitted to those data points the initial escape rate is likely to be underestimated.

The outgrowth of an escape variant and the subsequent replacement of the wild-type can be considered as a competitive growth between two populations:
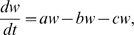
(6)

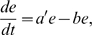
(7)where *w* is the wild-type and *e* is the escape variant. Cells infected with wild-type virus replicate at a net rate *a* and are killed by all CTL clones recognizing epitopes other than the escape epitope at rate *b*. The CTL clone recognizing the escape epitope kills wild-type cells at rate *c*. Cells infected with the escape variant replicate at a net rate *a*′ and are only killed by the CTL clones recognizing epitopes other than the escape epitope. For *c*>0, the escape variant gains a growth advantage as long as the fitness cost of the escape, *d* = *a*−*a*′, is not higher (i.e. *d*<*c*). As shown in Asquith et al. [12] this model can be fitted to longitudinal data of escape. This is done using the routine lmfit (http://sourceforge.net/projects/lmfit) based on the Levenberg-Marquardt algorithm to solve nonlinear least-squares problems. However, the fitting is likely to underestimate the true rates of escape as it is illustrated in Fig. 7.

### Killing Terms

We assume Michaelis-Menten kinetics for the killing of infected cells by CTL effector cells:
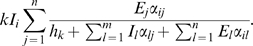
(8)This term takes into account the effector/target cell ratio of the cellular interaction: the total killing is proportional to infected cells at high effector cell densities and proportional to effector cells at high infected cell densities [28]. However, we also analyzed the outcome of other killing terms following different kinetics:
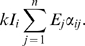
(9)Here, infected cells are killed by effector cells following simple mass-action kinetics. We mentioned that by increasing *h_k_* in Equation 8 we approach mass-action kinetics and the results are discussed.
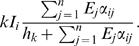
(10)Single saturation over CTL effector cells *E* is similar to the double-saturating term used in Equation 8: once the set-point is reached, 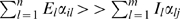, and Equation 8 approaches Equation 10.
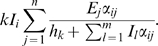
(11)Taking into account that CTL effector cells need some time to interact with infected cells before delivering their lethal hit, a saturation effect occurs as given in Equation 11 (see also reference [28]). We define 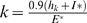, to have infected cells being killed at a maximal rate of 0.9 d^−1^ during the chronic phase of infection. I* and E* are the numbers of infected cells and CTL effector cells, respectively, in the steady-state in absence of escape. During the acute phase, we observe three types of dynamics: 1) For *h_k_*<5×10^7^, the infection is always cleared since an infected cell can be killed at a rate of 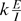 per day that can be enormous. 2) For 5×10^7^<*h_k_*<7×10^7^, a very rapid escape during acute infection can prevent clearance of the infection. 3) For *h_k_*>7×10^7^, we approach mass-action kinetics since *h_k_*>>*I* and the term becomes 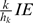.
